# Shortcomings of the controlled foreign company legislation concerning foreign tax refund systems

**DOI:** 10.1371/journal.pone.0341321

**Published:** 2026-02-02

**Authors:** Thomas Kollruss

**Affiliations:** Department of Business & Law, IU International University of Applied Sciences, Erfurt, Germany; Bina Nusantara University, INDONESIA

## Abstract

The aim of this study is to demonstrate that the CFC tax rules are legally ineffective in relation to those of foreign countries that operate a tax refund system at the shareholder level of the CFC (e.g., Malta). Under such a tax refund system, the CFC itself is highly taxed, but its shareholder receives a tax refund from the CFC’s country of residence upon profit distribution. Therefore, the research question is that the current CFC rules, owing to their legal structure, cannot effectively cover foreign countries of residence of the CFC that apply a tax refund system at the shareholder level. Using theoretical and legal research, this study shows that CFC taxation is largely ineffective in relation to foreign tax refund systems unless all of a CFC’s shareholders’ tax refunds are included in the calculation of the CFC’s tax burden. To illustrate this phenomenon, a comparative analysis of the CFC legislation in Germany, Austria and South Africa is conducted. The issue of foreign tax refund systems represents a significant challenge, as they have the potential to undermine the efficiency of CFC taxation as a pivotal instrument in the fight against tax avoidance. To solve this problem, a legal framework for the taxation of CFCs in countries with a tax refund system is being developed within this study to strengthen the taxation of CFCs and fight international tax avoidance. This study is a pioneering investigation of the legal efficiency of CFC taxation in the context of foreign jurisdictions that operate a tax refund system at the shareholder level of the CFC. It identifies the fundamental shortcomings of CFC tax law in the context of foreign tax refund systems and proposes legal solutions.

## Introduction

### Background and problem

CFC taxation is one of the most important instruments for combating cross-border tax avoidance. The EU’s Anti-Tax Avoidance Directive made CFC taxation mandatory in all EU Member States, with effects beginning in 2019. In addition, CFC taxation has been applied in other countries for a long period of time (the USA, Germany, China, Japan, Australia). The aim of CFC taxation is to combat the shifting of (mobile) income to low-tax foreign corporations, which generally have a tax shielding effect. As a result, CFC taxation means that the passive and low-tax income of the foreign intermediate company, the CFC, is attributed to the domestic shareholder for taxation purposes, irrespective of any distribution. Passive income encompasses various forms of revenue, including interest income, income derived from finance leases, receivables, cash and cash equivalents, and specific securities. The exact definition and scope of passive income are set out in the relevant CFC tax law. CFC taxation eliminates the tax shielding effect of the foreign company. The domestic taxpayer can no longer interpose a foreign corporation between itself and the source of income for tax purposes. However, CFC taxation requires that the passive income of the foreign intermediate company is taxed at a low rate. A calculation of its tax burden must be made.

A potential challenge arises when calculating the CFC’s tax burden in countries that apply a tax refund system. The tax refund mechanism built into these foreign countries provides for the reimbursement of a proportion of the tax paid by the CFC to its shareholder upon the distribution of profits. This suggests that when the tax refund at the shareholder level is included in the calculation of the CFC’s tax burden, the CFC’s income is effectively subject to low taxation. However, without special regulations, it is not possible to include the tax refund at the shareholder level in the calculation of the CFC’s tax burden, as companies and shareholders are separate taxpayers. The separation principle in tax law relating to investments in corporations also applies to CFC taxation.

This problem is illustrated via German CFC taxation as an example. The German provisions of Section 8 para. 5 sentence 2 AStG [1] only includes tax credits and tax refunds of shareholders of the foreign intermediate company with unlimited German tax liability in the tax burden calculation pursuant to Section 8 para. 5 AStG, but not from all shareholders. For example, tax refund claims of foreign shareholders of the intermediate company in which the German taxpayer does not hold an interest are de lege lata not included in the tax burden calculation of the foreign intermediate company. As a result, the effective income tax burden and any low taxation of the foreign intermediate company or passive income are incorrectly calculated. This considerably restricts the effectiveness of German CFC taxation in the case of foreign tax refund systems.

This finding indicates that CFC taxation does not apply if the CFC is based in a foreign country that operates a tax refund system at the shareholder level. Such foreign tax refund systems have the potential to undermine the effectiveness of CFC taxation. This is highly relevant because CFC taxation is one of the most important instruments for tackling cross-border profit shifting. Therefore, it is necessary to analyse the legal effectiveness of the current CFC tax rules in the context of foreign tax refund systems.

To demonstrate the limited legal effectiveness of CFC taxation with respect to foreign tax refund systems at the shareholder level, the CFC legislation of Austria and South Africa is also analysed. A comparative analysis (Germany, Austria, and South Africa) can then be used to assess the effectiveness of CFC tax rules and their necessary legal structure to cover those foreign tax refund systems.

### Objective of the study

The objective of the study is to demonstrate that the current CFC tax rules are legally ineffective in relation to those of foreign countries that operate a tax refund system at the shareholder level of the CFC (e.g., Malta). It provides an in-depth analysis of the legal factors in the current CFC tax law that cause the ineffectiveness of CFC legislation. Owing to their specific legal construction, the current CFC rules are generally unable to effectively cover foreign countries in which CFCs are residents and which apply a tax refund system at the shareholder level. With respect to this issue, the study highlights the general legal shortcomings of the CFC tax law. These weaknesses are because the low taxation of CFCs as a criterion for the application of CFC taxation is determined in isolation at the CFC level, without currently taking into account tax refunds granted to CFC shareholders by the foreign country in which the CFC is based. Therefore, the current CFC regulations are ineffective, as they focus solely on the CFC when calculating its effective tax burden. The present study investigates the issue in question and proposes a new legal framework that could be employed to strengthen CFC legislation.

Therefore, the objective of this study is to demonstrate that the CFC legislation has inherent design flaws with respect to foreign tax refund systems, particularly with respect to the determination of the low taxation of CFCs if only the tax refund claims of the domestic shareholders of the CFC are included in the determination of the tax burden of the CFC. The German CFC taxation is used as an example for the analysis and is presented in Section 8 para. 5 Sentence 2 AStG, which only includes tax refunds received by domestic shareholders of the CFC in the calculation of the CFC’s tax burden. Furthermore, Austrian and South African CFC tax rules have been incorporated into the analysis to demonstrate the broad basis of different legal taxation approaches. A comparative analysis then allows conclusions to be drawn as to how CFC regulations must be structured legally to effectively cover foreign tax refund systems.

### Main contribution of the study

This study shows for the first time that the CFC tax law is largely ineffective in relation to foreign countries that operate a tax refund system at the shareholder level of the CFC, unless all of a CFC’s shareholder tax refunds are included in the calculation of the CFC’s tax burden. This demonstrates that the legal construction and architecture of the current CFC laws are fundamentally incapable of addressing situations in which the CFC is based in a foreign country that applies a tax refund system at the shareholder level. It expands the existing academic literature on taxation. For the first time, it analyses the effectiveness of CFC rules in determining the low taxation of CFCs in the context of foreign tax refund systems and the effectiveness of CFC tax law in the context of foreign tax refund systems. Furthermore, the study highlights flaws in the legal construction and architecture of the CFC tax rules in relation to foreign tax refund systems and the calculation of the effective tax burden of CFCs. It proposes concrete solutions to strengthen the CFC’s tax rules. Thus, this study makes an important contribution, as CFC taxation is one of the most important instruments for combating cross-border profit shifting worldwide.

## Research question

The central research question of this study is that CFC taxation is legally ineffective in relation to foreign countries that operate a tax refund system at the shareholder level of the CFC (e.g., Malta). Owing to their legal design, the current CFC rules cannot effectively cover foreign countries of residence of the CFC, which apply a tax refund system at the shareholder level. In general, when calculating the tax burden of the CFC, CFC taxation does not apply if the relevant legal provisions do not include tax refunds received by the shareholders of the CFC or only includes tax refunds received by domestic shareholders of the CFC. Therefore, CFC taxation is largely ineffective in relation to foreign tax refund systems unless all of a CFC’s shareholders’ tax refunds are included in the calculation of the CFC’s tax burden. This is demonstrated via the example of German CFC taxation, which corresponds to that of all other EU Member States through the implementation of the EU Anti-Tax Avoidance Directive (BEPS).

The research question to be analysed, which posits that the CFC regulations may have potential shortcomings by failing to consider foreign tax refund systems, was derived analytically and conceptually in advance, taking into account the existing CFC tax rules. A comprehensive understanding of tax law and the CFC taxation system is essential, as the objective is not merely to apply tax law to a given situation but rather to identify a legal situation that is not currently covered by existing CFC rules through retrospective theoretical analysis. The relevant question or theory derived in this way, is therefore that the CFC tax law is principally ineffective in relation to foreign countries, which operate a tax refund system at the shareholder level of the CFC.

## Literature review and contribution of the study to the literature

The effectiveness of CFC taxation in relation to foreign tax refund systems has not been (extensively) studied in the academic literature. Importantly, the literature review cannot be comprehensive, as hardly any literature is available that addresses this topic. Therefore, the study at hand breaks new ground. In addition, new CFC taxation legislation was only introduced in the EU under the Anti-Tax-Avoidance Directive (ATAD; Council Directive (EU) 2016/1164 of 12 July 2016 [2]) with effect from 2019, meaning that hardly any literature is available in this regard, especially on special issues such as foreign tax refund systems and CFC taxation as well as the calculation of the tax burden of CFCs taking into account foreign tax refund systems. Kollruss (2019) addresses this problem [3]. He is the first to point this out in the literature. However, the literature has not yet addressed the issue and relevance of specific CFC tax burden calculation methods for foreign tax refund systems. Clifford (2019) addresses the financial and locational responses of multinationals to CFC rules but does not consider foreign tax refund systems in which CFCs may be residents [4]. She concludes that when effective, CFC rules have an effect on investment decisions. Paulus (2020) analyses the effects of CFC taxation on tax competition between states but does not consider foreign tax refund systems [5]. The statements in her study therefore do not relate to foreign tax refund systems and their effects on CFC taxation. Hansen et al. (2023) examine the effects of CFC taxation rules on the profit shifting behaviour of companies but do not address foreign tax refund systems [6]. They conclude that CFC taxation can generally restrict profit shifting if it is effective. Kuźniacki (2017) analyses tax avoidance by Polish shareholders through CFCs but does not specifically address foreign tax refund systems or the related problem of calculating the effective tax burden on passive income at the level of the CFC [7]. There is only a brief mention of Malta’s provision of a tax refund to CFC shareholders on distributions. Egger et al. (2015) address the impact of controlled foreign corporation legislation on real investment abroad by using a multidimensional regression discontinuity design [8]. However, they do not consider foreign tax refund systems or how they affect the decision of firms to invest in CFCs. Arnold (2019) examines the evolution of CFC rules but does not consider their relationship with foreign tax refund systems [9]. As a result, the study does not allow any conclusions to be drawn about the effectiveness of CFC taxation and its design in relation to foreign tax refund systems. Paulus (2022) analyses the effectiveness of the CFC taxation rules under the ATAD but addresses only the general rules here [10]. She does not analyse foreign tax refund systems or the effectiveness of the CFC rules in relation to such systems. Consequently, her analysis does not provide any indications as to whether the CFC taxation rules are effective in the case of foreign tax refund systems. Prettl et al. (2023) discuss multinational ownership patterns and CFC taxation [11]. However, they do not consider foreign tax refund systems such as Malta, which grant tax refunds in the event of profit distribution of the CFC at the level of the CFC’s shareholders. It is therefore not possible to conclude from this study whether the CFC regulations are effective for foreign tax refund systems and where their shortcomings might exist. Tokola (2022) discussed the changes in Finnish CFC taxation following the EU Anti-Tax Avoidance Directive but did not address foreign tax refund systems or their impact on the effectiveness of CFC taxation [12]. Öner (2024) analyses the rules of CFC taxation in light of their economic needs and from a tax policy perspective but does not address foreign tax refund systems or the effectiveness of CFC taxation in relation to such systems [13]. Bettens (2022) examines the taxation of CFCs in the context of the EU’s global minimum tax system (Pillar 2) and the strategic responses of taxpayers to these rules [14]. Foreign tax refund systems and how their use may affect the effectiveness of CFC taxation are not addressed. Gschossmann et al. (2024) discuss the location-related, financial and real reactions of multinational companies to the EU-wide implementation of CFC taxation by the ATAD [15]. However, they do not address foreign tax refund systems as the location of the CFC. Amberger et al. (2023) address international taxation in the context of the choice of legal form for FDI [16]. They do not deal specifically with CFC taxation or foreign tax systems with tax refunds in the case of profit distributions by the foreign subsidiary to its shareholders. However, there is no discussion of how foreign tax refund systems may affect the choice of location and the effectiveness of CFC taxation. The final report of the OECD BEPS project (2015) – Designing effective rules for controlled foreign companies, Action 3 – does not comment on the design of CFC taxation rules in relation to foreign tax refund systems [17].

## Methodology and justification of the research approach used

The aim of the present study is to show that the CFC tax law is legally ineffective in foreign countries that operate a tax refund system at the shareholder level. The subject of the study is legally based (power of tax law), so the research questions are pursued with a legal research methodology and theoretical analysis. The legal power of tax laws can be examined only via legal research methods. The same applies to the legal development of existing tax rules. Empirical research methods are not suitable in this context. Whether a tax norm can legally cover certain situations on the basis of its legal power cannot be determined by empirical analysis but only by legally based analysis and legal methodology. Theoretical analysis and mathematical modelling of the current tax effects of the respective tax legislation are part of the research process. In this way, shortcomings in existing legislation can be identified, and approaches to legal and legislative improvements to existing tax law can be derived. Legal and theoretical analysis is therefore well suited to determine the legal power of tax rules and to identify weaknesses at an early stage before tax losses occur. Empirical analysis cannot do this. It can only determine retrospectively whether a tax regime was ineffective on the basis of tax losses that have occurred, but it does not reveal where the legal weaknesses of tax regimes lie or how tax rules need to be improved in terms of their legal strength. Since the questions to be investigated are legally based, the methodological approach used here is suitable and appropriate.

Methodologically, the lack of effectiveness of CFC taxation under a foreign tax refund system is analysed theoretically. Theoretical and legal analysis is applied. Moreover, a theoretical case study is used where the CFC is located in Malta and the shareholder is a resident in Germany. This case study, supported by numerical examples, clearly shows how foreign tax refund systems at the shareholder level work and impede the effectiveness of CFC taxation. The relevant tax rules are analysed theoretically and quantitatively to reach reliable conclusions. With such a theoretical framework and experimental set-up, it is possible to analyse whether CFC tax rules, in this case, the German rules, have legal shortcomings in covering CFCs that are resident in foreign tax refund systems.

The fundamental problem is that CFC tax rules are based on the tax burden of the CFC itself in isolation and do, in principle, not take into account tax refunds received by the shareholders of the CFC from the foreign state of residence of the CFC when the low taxation of the CFC is determined. Therefore, CFC taxation systems may have a general conceptual weakness in relation to foreign tax refund systems. Furthermore, it is not sufficient to include only the tax refunds of domestic shareholders in the tax burden calculation of the CFC; however, the tax refunds of all shareholders of the CFC must be included in the tax burden calculation of the CFC to correctly determine the effective tax burden on the passive income of the CFC. This can be examined on the basis of German CFC taxation in the case of participation in a Maltese corporation. Malta grants the shareholders of the CFC a tax refund on distributions of up to 6/7 of the tax payable on the distributed profit. In addition, German CFC taxation, in this case, Section 8 para. 5  sentence 2 AStG, includes only the tax refunds of domestic shareholders in the calculation of the CFC’s tax burden but not the tax refunds received by its foreign shareholders. As a result, the effective tax burden of the CFC is incorrectly determined to be too high, and CFC taxation does not apply.

Furthermore, a comparative analysis of various legal CFC taxation models is performed. In addition to the German CFC rules, Austrian and South African regulations are included in the comparative analysis. Selecting these three countries enables different approaches to CFC taxation to be included and presented. For instance, the German CFC rules only include tax refunds from the foreign country of residence of the CFC in the calculation of the effective tax burden of the CFC in the event of profit distribution to its shareholders, to the extent that these refunds are attributable to shareholders that are resident in Germany. However, under Austrian CFC regulations, all tax refunds from the foreign country of residence of the CFC to its shareholders are included in the calculation of the effective tax burden of the CFC. The South African CFC regulations do not include tax refunds from the CFC’s country of residence to its shareholders when calculating the CFC’s effective tax burden concerning foreign tax years of controlled foreign companies ending before 31 December 2025. 

The selection of the three countries of Germany, Austria, and South Africa as the respective countries of residence of the CFC shareholder thus enables a comparative analysis and a statement on how CFC regulations must be legally designed to effectively cover foreign tax refund systems. The selection of these three countries is justified to demonstrate the legal effectiveness of CFC taxation in relation to foreign tax refund systems. This enables the exploration of a diverse range of legal CFC taxation settings, ranging from the partial inclusion of tax refunds to shareholders (as observed in Germany) to the comprehensive inclusion of all tax refunds (as implemented in Austria) and the noninclusion of such tax refunds (as evidenced in South Africa before 31 December 2025).

## Features of German CFC taxation

According to German CFC tax rules, the passive and low-taxed income of a CFC (foreign corporation) is attributed to the shareholder for tax purposes, regardless of distribution, provided the shareholder is subject to unlimited tax liability in Germany and holds, either directly or indirectly or together with related parties, more than 50% of the CFC’s shares. If the CFC’s income is primarily generated from capital investments, the minimum participation requirement for the German shareholder is reduced to 10% or less. The required participation of the German shareholder in the CFC must exist at the end of the CFC’s fiscal year. A CFC’s passive income includes, for example, interest income and income from finance leases, as well as certain capital gains resulting from holding, managing, maintaining or enhancing cash, receivables, securities and equity investments. The passive income of a CFC is taxed at a low rate if its income tax burden is less than 15%. To calculate this, the income taxes paid by the CFC on its passive income are divided by its passive income, as determined in accordance with German tax law (calculation). Any tax refunds received by (controlling) German shareholders of the CFC are taken into account when calculating its effective tax burden.

Sections 8 para. 5 sentence 2 and 12 para. 1 sentence 2 AStG of the German CFC taxation rules are intended to include “tax refunds”, which shareholders with unlimited German tax liability of a foreign intermediate company (CFC) receive from the country of residence of the CFC in the event of a profit distribution for determining low taxation of the CFC and CFC tax credits. These rules are directed against the Maltese corporate tax system and corresponding foreign taxation systems (“Lex Malta”). However, the effectiveness of these tax rules could be considerably reduced due to a legislative construction error in Sections 8 para. 5 sentence 2 and 12 para. 1 sentence 2 AStG. This legislative construction error lies in the fact that only claims that are granted to the taxpayer, i.e., the shareholder of the CFC with unlimited German tax liability (subject to the CFC income inclusion), in the event of a distribution by the country in which the CFC is domiciled are included in the tax burden calculation of the CFC with a reducing effect. Tax refund claims granted by the country of residence of the CFC to its foreign shareholders, in which the domestic taxpayer does not hold an interest, are currently not taken into account as a reduction when the low taxation of the CFC is determined. The same applies to the CFC tax credit volume pursuant to Section 12 para. 1 sentence 2 AStG.

The effective tax burden on a foreign CFC is thus determined in accordance with German CFC regulations as follows:


$$T_{GER}\;=\;\frac{Taxes\;paid\;by\;the\;CFC\;on\;its\;passive\;income-Total\;tax\;refund\;to\;German\;CFC\;shareholders}{Total\;passive\;income\;of\;the\;CFC\;determined\;in\;accordance\;with\;German\;tax\;law}$$

This means that a higher economic or effective income tax burden of the CFC is taken into account when its low taxation rate is determined, which is in line with Section 8 para. 5 AStG than actually exists. This is because the distribution of dividends by the CFC to its foreign shareholders also results in the latter receiving a tax refund and the foreign company’s passive income being taxed at an economically low level overall. The standardised nonrecognition of tax refunds granted to foreign shareholders of the CFC means that the (effective) income tax burden of the CFC is calculated to be too high, even though it is actually lower. Similarly, according to Section 12 para. 1 sentence 2 AStG, de lege lata, too much foreign corporate income tax is credited against the German income or corporate income tax of the unlimited taxpayer (shareholder of the CFC) if this provision is applied.

Accordingly, Section 8 para. 5 sentence 2 AStG and the subsequent Section 12 para. 1 sentence 2 AStG are likely to contain a significant legislative design error, as currently, only tax refunds to shareholders with unlimited German tax liability (taxpayers) of the CFC are taken into account, not to foreign shareholders of the CFC.

As a result, German CFC taxation does not apply if foreign shareholders hold a certain amount of the nominal capital of the CFC (at least 1/3), even though the passive income attributable to shareholders with unlimited German tax liability is (effectively) a low tax. This will be investigated below as part of an analysis, including a case study.

## Theoretical analysis (German CFC taxation)

The special feature of some foreign tax refund systems, particularly Malta, is that the income tax burden and corporate tax payment of the CFC (corporation) domiciled there remain unchanged. See Kollruss (2017) [18] and Vroom (2010) [19]. For example, a Maltese corporation and subsidiary is generally subject to a Maltese corporate income tax of 35% on its income. This tax burden and tax payment by the Maltese corporation to the Maltese tax authorities does not change if the shareholder of the Maltese corporation, another person, receives a tax refund from the Maltese tax authorities of up to 6/7 of the tax burden on the distributed profit.

This means that the tax burden of the CFC itself remains unaffected by any tax refund at the level of its shareholders. In principle, CFC taxation is based on the tax burden of the CFC in isolation (Section 8 para. 5 sentence 1 AStG). Section 8 para. 5 sentence 2 AStG is intended to overcome this and includes tax refunds at the shareholder level for determining the tax burden of the CFC. However, Section 8 para. 5 sentence 2 AStG de lege lata does not go far enough, as only tax refunds from shareholders of the CFC with unlimited German tax liability are taken into account to calculate the tax burden of the CFC but not tax refunds that foreign shareholders of the CFC receive or can claim.

This is clearly demonstrated by the wording of Section 8 para. 5 sentence 2 AStG:

“***Claims granted to the taxpayer*** *by the state or territory of the foreign company in the event of a distribution of profits by the foreign company [...] must be included in its tax burden calculation*”.

The taxpayer is the unlimited German taxpayer within the meaning of Section 7 para. 1 sentence 1, Section 13 para. 1 AStG, which is subject to German CFC taxation inclusion. Foreign shareholders of the CFC, in which the German taxpayer does not hold a stake, are not subject to the provisions of Section 8 para. 5 sentence 2 AStG. Thus, de lege lata, tax refund claims received by foreign shareholders of the CFC cannot be included in the tax burden calculation of the CFC in accordance with Section 8 para. 5 sentences 2 and 1 AStG.

Therefore, Section 8 para. 5 sentence 2 AStG falls short. This provision concerns the determination of the low taxation of the CFC. The issue here is whether the passive income of the CFC is subject to an income tax rate of less than 15% (low taxation). See also [20]. This is still the factual level of CFC taxation [21] only then, as a legal consequence, the passive and low-tax income for which the CFC is the intermediate company, included as a taxable CFC income inclusion amount (pro rata) for shareholders with unlimited German tax liability.

Whether the passive income attributable to the unlimited taxpayer in the legal consequence is taxed at a low level in isolation is irrelevant. Section 8 para. 5 AStG and sentence 2 of this provision relate upstream to the tax burden on passive income at the CFC itself. See also BMF 2023 [22]. The passive income is not yet allocated to the taxable shareholders of the CFC. Thus, Section 8 para. 5 sentence 2 AStG falls short if it seeks to determine the economic or effective tax burden of the passive income at the level of the CFC, but only tax refund claims of shareholders with unlimited German tax liability within the meaning of Section 7 para. 1, Section 13 para. 1 AStG and not of foreign shareholders or all other shareholders of the CFC, even though they receive corresponding tax refund claims in the event of a profit distribution, and the passive income of the foreign intermediate company is therefore subject to lower effective taxation overall. Not considering tax refunds to foreign shareholders of the CFC when determining the tax burden on the passive income of the CFC, its effective tax burden is overstated de lege lata.

Section 8 para. 5 sentence 2 AStG appears to contain a logical error in addition to the legislative design error. The provision does not consider that it is a matter of determining the tax burden of the CFC with the passive income in toto (factual level; [22,23]). Section 8 AStG and Section 8 para. 5 AStG are concerned with whether the CFC is an intermediary company. The determination of the low taxation of its passive income is a prerequisite for this. See also [23]. The determination of a tax burden with regard to the passive income (proportionately) attributable to the unlimited German taxpayer in the legal consequence does not occur in the system of CFC taxation. This is further evidenced by Section 12 para. 1 sentence 1 AStG. According to this provision, the income taxes that have actually been levied on the passive and low-tax income at the expense of the CFC are credited against the income tax of the unlimited German taxpayer.

## Theoretical case study (German CFC taxation)

**Fig 1:**
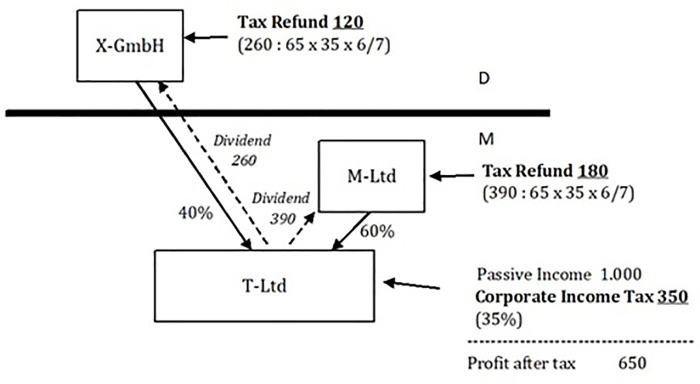
Tax Refund at the shareholder level (own illustration).

X-GmbH, a German corporation that has unlimited German tax liability, holds 40% of the nominal capital and voting rights of the Maltese corporation T-Ltd [Fig 1]. The other shareholder is the Maltese company M-Ltd., which holds 60%. X-GmbH does not hold an interest in M-Ltd. Both are unrelated persons and are not related parties pursuant to Section 1 para. 2 and Section 7 para. 4 AStG. The Maltese T-Ltd only generates passive income of an investment nature within the meaning of Section 13 para. 2 AStG. Its financial year corresponds to the calendar year. In the relevant financial year, its passive income is 1,000. The Maltese corporate income tax rate is 35%. Upon distribution, the shareholders of the Maltese T-Ltd. are entitled to a tax refund of 6/7 of the tax charged on the distributed profit. In the relevant financial year in which the aforementioned passive income is realised, an advance profit distribution is made to the shareholders of T-Ltd in the maximum possible amount. The escape from CFC taxation pursuant to Section 8 para. 2 AStG is not provided for Maltese T-Ltd.

When it comes to the tax refund that a shareholder of a Maltese corporation receives upon profit distribution, the calculation is generally as follows:


$${Tax\;Refund}_{Shareholder}=\;\frac{Total\;gross\;dividend\;distributed\cdot Shareholder\;participation\;ratio\;}{\text{6}\text{5}}\;\cdot \text{3}\text{5}\;\cdot \;\frac{\text{6}}{\text{7}}$$

This calculation formula can be interpreted as follows: The shareholder receives a tax refund equivalent to six-sevenths of the tax levied on the distributed profit. The Maltese corporate income tax rate is 35%. With a pre-tax profit of 100, the distributable profit of the company or the maximum gross dividend is 65. Multiplying this by the shareholder’s participation ratio gives the proportionate gross dividend. To calculate the Maltese corporate income tax levied on the proportionate dividend, first divide this by 65 and then multiply by 35. The Maltese tax refund for the respective shareholder is obtained by multiplying this amount by 6/7.

In accordance with Section 8 para. 5 sentence 2 AStG, only the tax refund received by X-GmbH, which has unlimited German tax liability, is to be included in the tax burden calculation of the CFC T-Ltd (120). De lege lata, the tax refund that the foreign shareholder M-Ltd of T-Ltd receives, here 180, is not included in the tax burden calculation of the CFC. In line with Section 8 para. 5 sentences 2 and 1 AStG, the tax burden on the passive income of the CFC T-Ltd is (350–120): 1,000 × 100 = 23%. This means that the income for which the foreign company T-Ltd is the intermediary company, the CFC, the passive income, is not subject to an income tax of less than 15%. Therefore, German CFC taxation does not apply to X-GmbH, which has unlimited German tax liability, owing to the lack of low taxation of the CFC.

However, the passive income attributable to X-GmbH, which has unlimited German tax liability, is effectively taxed at a low rate: 1,000 × 0.4 × 0.35–120 = 20. It is subject to an effective tax burden of 20 in relation to pro rata passive income of 400, which corresponds to an effective tax burden of 5%.

If, de lege ferenda, the tax refunds of foreign shareholders or all shareholders of the CFC were also correctly included in the tax burden calculation of the CFC, this would result in a tax burden of 5% on the passive income of the CFC and thus effective low taxation (350–120–180): 1,000 × 100 = 5%. CFC taxation applies.

This makes it clear that the legislative construction error of Section 8 para. 5 sentence 2 AStG according to which only tax refunds received by its shareholders with unlimited German tax liability within the meaning of Section 7 para. 1, Section 13 para. 1 AStG (and not all shareholders of the CFC) are included in the tax burden calculation of the CFC, which leads to the fact that the CFC taxation is not applicable because of the lack of a correct determination of the effective tax burden of the CFC’s passive income.

The German tax legislature will therefore have no choice but to extend Section 8 para. 5 sentence 2 AStG soon aims to include the tax refunds of all shareholders of the CFC in its tax burden calculation if it wishes to ensure the effectiveness of the CFC taxation provisions.

## Theoretical-quantitative analysis

In this section, the relevant German CFC tax law provisions are transformed into a mathematical equation. This equation is used to analyse the effects of foreign tax refund systems on the calculation of the low taxation of foreign CFCs. A mathematical equation can be used to determine the threshold value beyond which the CFC regulation becomes legally ineffective in the context of foreign tax refund systems.

The mathematical equation makes it possible to filter out the shareholding ratio of foreign shareholders in the nominal capital of the (Maltese) CFC, above which the German CFC taxation does not apply to shareholders with unlimited tax liability due to the noninclusion of tax refunds pursuant to Section 8 para. 5 Sentence 2 AStG received by foreign shareholders of the CFC. It is also assumed that the Maltese 6/7 tax refund to shareholders is relevant.

The passive income (P_E_) of the Maltese company is subject to corporate income tax (s_k_ = 35% or 0.35). With respect to the nominal capital participation (β) of shareholders with unlimited German tax liability of the Maltese company, the tax refund, which amounts to 6/7 of the tax charged on the (proportionate) distributed profit of the Maltese company, is included in the tax burden calculation of the Maltese company in accordance with Section 8 para. 5 sentence 2 AStG. The distribution ratio of the Maltese company is referred to as α. By definition, it can assume the following values:


0 < α < 1.

α = 1 means that the Maltese company, the CFC, distributes its entire profit after tax to its shareholders. Considering the reduced inclusion of the tax refund of the Maltese company’s shareholders with unlimited German tax liability in the CFC’s tax burden calculation, the CFC's total income tax burden must not be less than 15% (s_g_ > 0.15). This results in the general determination equation:


$$P_{E}\;\cdot s_{K}-\;[P_{E}\;\cdot \left({\text{1}-s}_{K}\right)\;\cdot \;\alpha \;\cdot \;\frac{\text{3}\text{5}}{\text{6}\text{5}}\;\cdot \;\frac{\text{6}}{\text{7}}\cdot \;\beta \;\geq P_{E}\;\cdot \text{0},\text{1}\text{5}$$

A profit distribution ratio of 100% is assumed (α = 1). S_k_ is 0.35. These values are used in the general determination equation. Furthermore, both sides of the equation can be divided by P_E_, resulting in the following simplified form:


$$\text{0},\text{3}\text{5}-\;[(\text{0},\text{6}\text{5})\;\cdot \;\frac{\text{3}\text{5}}{\text{6}\text{5}}\;\cdot \;\frac{\text{6}}{\text{7}}\cdot \;\beta \;\geq \text{0},\text{1}\text{5}$$

When further resolved, this results in


$$\begin{array}{c} 0,3\;\beta \;\leq \;0,2\;or\;\beta \;\leq \;\frac{2}{3} \end{array}$$

This means that the shareholding of an unlimited German taxpayer (in this case X-GmbH) in the nominal capital of the Maltese company may not exceed 2/3 so that the German CFC taxation does not apply, taking into account the current CFC’s tax burden calculation in accordance with Section 8 para. 5 sentences 2 and 1 AStG.

To summarise, if foreign shareholders hold at least 1/3 of the nominal capital of the Maltese CFC, German CFC taxation does not apply. This makes it easy to avoid CFC taxation in relation to Maltese subsidiaries if, in addition to the German shareholder, there are foreign companies or foreign shareholders in this company that hold at least 1/3 of the nominal capital of the CFC and the taxpayer with unlimited German tax liability does not have a stake in these foreign shareholders. This means that the German taxpayer can even control the Maltese CFC (more than 50% of the shares in the nominal capital and voting rights) without the German CFC taxation coming into effect if, in addition to this taxpayer, foreign shareholders hold at least 1/3 of the nominal capital of the Maltese CFC.

This makes it clear that the “Lex Malta” (Section 8 para. 5 sentence 2 AStG) in its current version is not expedient due to its legislative design flaw. Therefore, the German tax legislature should improve this provision soon if it wishes to guarantee the effectiveness of German CFC taxation in relation to special foreign tax refund systems (in particular, Malta).

## Austrian CFC taxation and comparative analysis

Under the Austrian CFC tax rules, the foreign corporation (CFC) is subject to low taxation if its tax burden abroad does not exceed 12.5% (§ 10a (3) of the Austrian Corporate Income Tax Act/A-CITA). For the purpose of calculating the effective tax burden of the CFC, the tax actually paid by the CFC abroad must be divided by the total passive income of the CFC, which is determined in accordance with Austrian tax regulations.

The regulation of the passive income of low-taxed entities (VO-Passiveinkünfte, BGBl. II Nr. 21/2019) provides additional information on how to determine the taxes actually paid by the CFC. The following provisions are laid down in Section 1 (3) No. 4 VO-Passiveinkünfte concerning foreign tax refund systems:

‘*Taxes refunded to the shareholders of the CFC are not considered to have been paid by the CFC. If a tax refund can only be claimed in a subsequent fiscal year, this must be taken into account when calculating the average tax* burden of the CFC’.

In contrast to the German CFC tax rules, the Austrian CFC regulations include all tax refunds from the foreign country of residence of the CFC in the calculation of the effective tax burden of the CFC. Notably, tax refunds to domestic and foreign shareholders of the CFC are also taken into account when the effective tax burden of the CFC is calculated. This means that any tax refunds received by the shareholders of the CFC reduce the CFC’s effective tax burden for Austrian tax purposes.

Furthermore, tax refunds to CFC shareholders must be considered when determining the effective tax burden of the CFC, provided that they are legally feasible in principle and can be claimed in subsequent financial years through actual distributions.

To summarise, Austrian CFC regulations determine the effective tax burden (*T*_*AT*_) of each CFC as follows, with this calculation being made for each calendar year (tax assessment period):


$$T_{AT}=\;\frac{Taxes\;paid\;by\;the\;CFC\;on\;its\;passive\;income-Total\;tax\;refund\;to\;all\;CFC\;shareholders}{Total\;passive\;income\;of\;the\;CFC\;determined\;in\;accordance\;with\;Austrian\;tax\;law}$$

The legal effectiveness of Austrian CFC taxation can now be verified via the figures from the theoretical case study above.

In this scenario, the CFC generates a passive income of 1.000. The Maltese CFC paid a tax rate of 35% on this income (350). Accordingly, an Austrian corporation holds 40% interest in the Maltese CFC (having more than 50 % of the voting rights in the CFC) and receives a tax refund of 120. Furthermore, a foreign corporation holds 60% interest in the Maltese CFC and receives a tax refund of 180. In total, Malta, as the country of residence of the CFC, refunds 6/7 of the taxes paid by the CFC in the event of a distribution to its shareholders (350 × 6 : 7 = 300). The Maltese CFC’s after-tax profit (1.000–350 = 650) is fully distributed to shareholders.

According to Austrian CFC tax law, the effective tax burden of the CFC is now calculated as follows:


$$T_{AT}=\frac{\text{3}\text{5}\text{0}-(\text{1}\text{2}\text{0}+\text{1}\text{8}\text{0})}{\text{1}.\text{0}\text{0}\text{0}}=\;\frac{\text{5}\text{0}}{\text{1}.\text{0}\text{0}\text{0}}=\text{5}\%<\text{1}\text{2}.\text{5}\%\;$$

This means that the CFC is subject to low taxation, as its effective tax burden is only 5% and lower than 12.5% (low taxation). Therefore, Austrian CFC taxation applies, and 40% of the CFC’s passive income is attributed to the Austrian corporation as a shareholder of the CFC for Austrian tax purposes.

Thus, the Austrian CFC regulations are very effective in countering foreign tax refund systems. This is because they include all tax refunds to CFC shareholders in the calculation of the CFC’s effective tax burden, not just those received by Austrian CFC shareholders.

The situation is different with respect to German CFC regulations, which, as shown above, only include tax refunds received by German resident shareholders of the CFC in the calculation of the effective tax burden of the CFC. This can be demonstrated via a comparative calculation. According to German CFC tax law, the tax threshold of CFC low taxation is defined as a tax burden on the CFC of less than 15%. For German tax purposes, the effective tax burden of the CFC (*T*_*GER*_) is calculated as follows:


$$T_{GER}=\;\frac{Taxes\;paid\;by\;the\;CFC\;on\;its\;passive\;income-Total\;tax\;refund\;to\;German\;CFC\;shareholders}{Total\;passive\;income\;of\;the\;CFC\;determined\;in\;accordance\;with\;German\;tax\;law}$$

Applying the figures from the theoretical case study above results in the following effective tax burden on the CFC for German tax purposes:


$$T_{GER}=\frac{\text{3}\text{5}\text{0}-\text{1}\text{2}\text{0}}{\text{1}.\text{0}\text{0}\text{0}}=\;\frac{\text{2}\text{3}\text{0}}{\text{1}.\text{0}\text{0}\text{0}}=\text{2}\text{3}\%>\text{1}\text{5}\%\;$$

According to German CFC regulations, the effective tax burden on CFCs is calculated as too high, despite passive income being subject to an effective tax rate of just 5%. German CFC taxation therefore does not apply. This discrepancy arises from a legal construction flaw in the German CFC tax rules, which only include tax refunds to German shareholders of the CFC in the calculation of the effective tax burden of the CFC. However, tax refunds to all CFC shareholders are not taken into account.

The situation is different under the Austrian CFC tax law. These regulations include all tax refunds to shareholders of the CFC as a reduction in the CFC’s effective tax burden, thereby accurately reflecting the calculation of the CFC’s actual tax burden on passive income.

The key finding of the comparative analysis of Germany and Austria is that CFC regulations must include all tax refunds to all CFC shareholders, not just domestic ones, when calculating the effective tax burden on passive CFC income if they are to be effective against foreign tax refund systems. If this is not done, as the German CFC regulations show, the effective tax burden of the CFC is calculated too high, and CFC taxation does not apply, even though the passive income is actually taxed at a low rate. Therefore, affected countries must adjust their CFC regulations to include tax refunds for all CFC shareholders when determining the CFC’s low taxation.

The CFC’s tax regulations cannot be effective in relation to foreign tax refund systems if they do not take into account any tax refunds to the shareholders of the CFC when determining the low taxation or effective tax burden of the CFC. This is exemplified by the CFC regulations in South Africa applicable for CFC tax years ending before 31 December 2025. This issue will be addressed in a subsequent section.

## South African CFC taxation and comparative analysis

For South African CFC tax purposes, the passive and low-tax income of a controlled foreign corporation (CFC) is attributed to its South African shareholder if the latter holds at least a 10% interest in the CFC (Section 9D of the Income Tax Act 58 of 1962). However, the CFC’s passive income abroad is not low tax if it would be subject to a tax burden of at least 67.5% if the CFC were a South African company. This is the high-tax exemption. The corporate income tax rate for a South African corporation is currently 27%. This means that a CFC is subject to low taxation if its effective tax burden on its passive income abroad is less than 18.225% (27% x 0.675).

To determine the effective tax burden on the CFC, the taxes actually paid by the CFC on its passive income must be divided by the CFC’s total passive income, as determined in accordance with South African tax laws. The effective tax burden of each CFC is calculated on a standalone basis. South Africa’s CFC tax rules do not include any provisions stipulating that tax refunds from a CFC’s country of residence to its shareholders must be taken into account when calculating the CFC’s effective tax burden. This applies to CFC tax years ending before 31 December 2025. This is a key difference between (the old) South African CFC tax rules and those in effect in Germany and Austria. In the latter cases, tax refunds to CFC shareholders are partially or fully included in the calculation of the CFC’s effective tax burden.

In summary, the effective tax burden of a CFC (*T*_*SA*_) under the South African CFC tax rules is calculated as follows concerning CFC tax years ending before 31 December 2025:


$$T_{SA}=\;\frac{Taxes\;actually\;paid\;by\;the\;CFC\;on\;its\;passive\;income}{Total\;passive\;income\;of\;the\;CFC\;determined\;in\;accordance\;with\;South\;African\;tax\;law}$$

Since a tax refund from the CFC’s foreign country of residence to the CFC shareholders does not affect the amount of taxes actually paid by the CFC and since there are no special provisions in South African CFC tax law for including tax refunds at the level of CFC shareholders when calculating the CFC’s effective tax burden (concerning CFC tax years ending before 31 December 2025), the CFC’s tax burden is incorrectly calculated as higher than it actually is, although the CFC’s passive income is subject to low taxation from an economic perspective.

Using the data from the theoretical case study above, the following tax burden on the CFC arises pursuant to the South African CFC tax rules before 2026:


$$T_{SA}=\frac{\text{3}\text{5}\text{0}}{\text{1}.\text{0}\text{0}\text{0}}=\;\text{3}\text{5}\%>\text{1}\text{8}.\text{2}\text{2}\text{5}\%\;$$

These findings clearly indicate that South African CFC tax legislation before 2026 is not adequately designed to effectively cover foreign tax refund systems. The reason behind this is that tax refunds at the level of CFC shareholders are not taken into account when determining the effective tax burden of CFCs. This approach contrasts with the German and Austrian CFC regulations, which consider tax refunds at the level of CFC shareholders to a certain extent or in full. This issue has been noted by South African tax legislators. The Taxation Laws Amendment Bill 2025 seeks to incorporate tax refunds at the level of the CFC shareholders into the calculation of the effective tax burden of CFCs (concerning tax years of the CFC ending on or after 31 December 2025). However, many countries worldwide still have CFC regulations in force that do not include the necessary rules stipulating that tax refunds at the shareholder level must be taken into account when determining the effective tax burden of CFCs. The issue under discussion arises from the fact that the CFC tax law traditionally focuses solely on the CFC when its effective tax burden is determined. Furthermore, tax law applies the separation principle to shareholdings in corporations. Germany and Austria incorporated corresponding provisions into their CFC tax laws, including tax refunds at the CFC shareholder level in the calculation of the CFC’s effective tax burden.

Importantly, a similar problem exists in the European Union’s CFC rules, which are set out in the Anti-Tax Avoidance Directive (Art. 7 and 8 ATAD; Council Directive (EU) 2016/1164 of 12 July 2016). In this instance, there are no specific provisions that permit tax refunds at the level of the CFC shareholders to be included (as a reduction) in the calculation of the CFC’s effective tax burden. Consequently, ATAD CFC tax provisions are generally ineffective in relation to foreign tax refund systems taking place at the shareholder level of the CFC.

Therefore, the comparative analysis in this study revealed a problem of general relevance regarding the legal effectiveness of CFC tax law in relation to foreign tax refund systems. The findings and conclusions of the comparative analysis are summarised below [Table 1].

**Table 1:** Comparative analysis of CFC legislation in Germany, Austria, and South Africa and implications for the legal design of CFC tax law with regard to its effectiveness in relation to foreign tax refund systems (own illustration).

Country	*Germany*	*Austria*	*South Africa*
***CFC tax rules which cover foreign tax refund systems that grant tax refunds to CFC shareholders***	Corresponding regulations exist in the CFC tax law (Section 8, para. 5 AStG). However, only tax refunds received by CFC shareholders resident in Germany from the foreign country of residence of the CFC are included in the calculation of the effective tax burden of the CFC.	Corresponding regulations exist in the CFC tax law (§ 10a (3) A-CITA; Section 1 (3) No. 4 VO-Passiveinkünfte). All tax refunds paid by the foreign country of residence of the CFC to the shareholders of the CFC, regardless of whether they are domestic or foreign shareholders, are included in the calculation of the effective tax burden of the CFC.	The South African CFC tax law does not contain any provisions regarding foreign tax refund systems at the level of the CFC shareholders. This concerns tax years of the CFC ending before 31 December 2025.
***Legal effectiveness of the CFC regulations***	The effectiveness of CFC taxation rules is limited, as the actual tax burden on CFC income is not determined. Therefore, the existing CFC rules are only partially effective against foreign tax refund systems.	Full effectiveness of CFC regulations in relation to foreign tax refund systems.	CFC taxation is not effective in relation to foreign tax refund systems at the level of the CFC shareholders.
***Need to adjust the CFC tax law***	The current CFC tax rules should be amended to ensure that all tax refunds to CFC shareholders, whether domestic or foreign, are considered when calculating the CFC’s effective tax burden.	It is not necessary to adjust CFC regulations with regard to their effectiveness in relation to foreign tax refund systems. However, the regulations must be designed in accordance with EU law (Art. 49 TFEU), as tax refunds to shareholders may only be taken into account in reducing the effective tax burden of the CFC if the CFC shareholders have actually received them.	Adjustment of the CFC tax law is imperative with regard to the inclusion of tax refunds to CFC shareholders in the calculation of the effective tax burden of the CFC. A legal development in this regard is the Taxation Laws Amendment Bill 2025 including tax refunds at the shareholder level of the CFC when calculating the effective tax burden of the CFC.This will come into operation for CFC tax years ending on or after 31 December 2025.
***Final conclusion for the legal structure and design of the CFC tax law***	The CFC tax rules must include special provisions for tax refunds granted by the foreign country of residence of the CFC to its shareholders, when determining the CFC’s effective tax burden. All CFC shareholders’ tax refunds must be taken into account, regardless of whether they are domestic or foreign. To ensure compliance with EU law and constitutional law (ability-to-pay principle), the calculation of the CFC’s tax burden should only include tax refunds actually received by its shareholders.		

## Summary and conclusion

### Theoretical contributions and findings

The present CFC tax rules are generally designed in such a way that they concern only the foreign company (CFC) and its tax burden, with no consideration given to tax refunds at the shareholder level of the CFC. Owing to this specific legal construction, the CFC tax law has a general weakness with respect to foreign tax refund systems, which refund a substantial part of the tax paid by the foreign company (CFC) to the shareholders of the CFC when profits are distributed. Therefore, under the current CFC regulations, the CFC’s tax burden is overstated, as tax refunds from the CFC’s country of residence to the CFC’s shareholders are not included in the calculation of the CFC’s tax burden. From an economic perspective, the income of the CFC is subject to a low tax rate. However, CFC taxation does not apply, as the rules are generally based on the tax burden of the foreign company (CFC) and do not take into account tax refunds at the shareholder level. Therefore, the current CFC tax rules are legally ineffective in relation to those of foreign countries that operate a tax refund system at the shareholder level of the CFC (e.g., Malta). These important findings have been derived through theoretical and legal analysis.

Moreover, the study shows that CFC taxation is largely ineffective in relation to foreign tax refund systems unless all of a CFC’s shareholders’ tax refunds are included in the calculation of the CFC’s tax burden. For CFC taxation to be effective, it is not sufficient to include only tax refunds received by domestic shareholders of the CFC in the calculation of the tax burden of the foreign company (CFC). The German CFC tax regime offers a practical illustration of this solution. However, theoretical and legal analysis has demonstrated that this legal approach is inadequate for ensuring the effective coverage of foreign tax refund systems.

These findings are confirmed by a comparative analysis of CFC tax law in Germany, Austria and South Africa. These three countries have different CFC taxation rules in force with respect to foreign tax refund systems. Under the German CFC tax law, only tax refunds to CFC shareholders resident in Germany are taken into account when determining the effective tax burden of the CFC. However, this is not the case for foreign CFC shareholders. Consequently, it is evident that the German CFC tax legislation is not adequate to ensure the full effectiveness of CFC taxation in relation to foreign tax refund systems. The situation is different from that of Austrian CFC taxation, which includes tax refunds from the foreign country of residence of the CFC to domestic and foreign shareholders of the CFC to determine the effective tax burden of the CFC. Only this tax approach is appropriate for enabling effective CFC taxation in relation to foreign tax refund systems. The South African CFC taxation regulations, applicable to CFC tax years ending before 31 December 2025, do not include tax refunds at the level of the CFC shareholders when calculating the effective tax burden of CFCs. The focus is solely on the tax payments of CFCs. This approach to taxation is not only inappropriate but also ineffective in relation to foreign tax refund systems. Consequently, South Africa is including tax refunds for CFC shareholders in the calculation of the effective tax burden of CFCs through the Taxation Laws Amendment Bill 2025. This new provision applies to CFC tax years ending on or after 31 December 2025.

The legal effectiveness of CFC taxation in relation to foreign tax refund systems, such as that employed by Malta, is contingent upon the inclusion of the tax refunds of all shareholders of the CFC in the calculation of the CFC’s tax burden. This legal approach is essential for ascertaining the actual or effective tax burden of the CFC’s income. The issue of foreign tax refund systems represents a significant challenge, as they have the potential to undermine the efficiency of CFC taxation as a pivotal instrument in the fight against tax avoidance.

### Practical implications

Accordingly, states must include provisions in their CFC taxation rules that require tax refunds received by the CFC’s shareholders to be included in the CFC’s tax burden calculation. This is the most effective way to ensure the effectiveness of CFC taxation in relation to foreign tax refund systems. It is recommended that all tax refunds received by shareholders of a CFC be included in the CFC’s tax burden calculation, not only those of domestic shareholders. This approach ensures the effectiveness of CFC taxation when confronted with foreign tax refund systems. This approach must be implemented to be effective in the fight against CFC tax avoidance and in line with the objectives of OECD BEPS Action 3.

Furthermore, each country reserves the right to adjust its CFC tax legislation at any time to determine the effective tax burden on CFCs. The Anti-Tax Avoidance Directive does not preclude these actions, as it merely establishes a minimum standard for EU countries and reserves the right to implement stricter regulations (Art. 3 ATAD). The global minimum tax does not preclude this possibility either. The global minimum tax is applicable subsequent to CFC taxation and addresses a distinct taxation issue. Additionally, a considerable number of countries have not yet implemented the global minimum tax. The solution to modify the calculation of the effective tax burden of a CFC in the respective national tax legislation, and to incorporate all direct and indirect shareholders of the CFC with their tax refunds received from the foreign country of residence of the CFC in the calculation of the effective tax burden of the CFC, is therefore feasible in legislative and administrative terms.

To strengthen the taxation of CFCs and combat international tax avoidance, an appropriate legal framework for the taxation of CFCs in countries with a tax refund system has been developed and discussed in this study. To this end, de lege ferenda, the tax refund claims of all CFC shareholders should be included when calculating the effective tax burden of the CFC on its income.

### Limitations and further research directions

The study analysed how foreign tax refund systems affect the taxation of controlled foreign corporations (CFCs) under the current CFC rules. It constitutes a pioneering investigation into the legal power of CFC taxation within the context of foreign jurisdictions that operate a tax refund system at the shareholder level of the CFC. This study identifies the fundamental shortcomings of the current CFC tax law in the context of foreign tax refund systems and proposes legal solutions for future tax legislation. In this way, the study makes an important contribution to strengthening the legal power of CFC taxation and to combating tax avoidance through profit shifting by CFCs. This study focused on one of numerous issues: foreign tax refund systems and CFC taxation. However, there are other potential shortcomings of CFC taxation that should be analysed in future studies. One unresolved issue is the application of CFC taxation to orphan structures, i.e., legal entities such as foreign foundations that have no shareholders. The CFC rules of many countries currently fall short in this regard, as there are no specific rules to effectively cover these situations. Additionally, the EU’s CFC taxation under the Anti-Tax Avoidance Directive (BEPS) does not address this issue. Another issue not yet fully analysed is how to address qualification conflicts within CFC taxation. For example, CFC taxation can currently be avoided in a number of situations where the CFC uses hybrid financial instruments. Thus, further research is needed in this area to improve the use of CFC taxation rules.
